# Acupuncture with twirling reinforcing and reducing manipulation shows a control of hypertension and regulation of blood pressure-related target brain regions in spontaneously hypertensive rat: a preliminary resting-state functional MRI study

**DOI:** 10.3389/fnins.2023.1161578

**Published:** 2023-05-26

**Authors:** Yin-Yin Li, Ji-Peng Liu, Shu-Feng Shi, Ke-Zhen Yang, Yu Gong, Jiao Sun, Qi Xie, Xiao-Li Wu, Qing-Guo Liu, Meng Xu

**Affiliations:** ^1^School of Acupuncture-Moxibustion and Tuina, Beijing University of Chinese Medicine, Beijing, China,; ^2^Department of Tuina, Beijing University of Chinese Medicine Third Affiliated Hospital, Beijing, China

**Keywords:** acupuncture, twirling reinforcing and reducing manipulation, spontaneously hypertensive rats, resting-state functional MRI, central mechanism

## Abstract

**Aim:**

To observe the effects of acupuncture manipulations on blood pressure and brain function in spontaneously hypertensive rats and elucidate the anti-hypertensive effect of the manipulations’ central mechanism.

**Methods:**

This study used acupuncture twirling reinforcing, acupuncture twirling reducing, and acupuncture twirling uniform reinforcing-reducing manipulations to act on the bilateral TaiChong point of rats. The depth of acupuncture was 1.5–2 mm, and twisting was performed at a frequency of 60 times/min within ±360° for 3 min, followed by the needle being retained for 17 min. Functional magnetic resonance imaging was performed at the end of the intervention. Regional homogeneity and amplitude of low-frequency fluctuations were used to assess the differences in brain regions in each group of rats, and the core brain region (left hypothalamus) among the differential brain regions was selected as the seed for functional connectivity analysis.

**Results:**

(1) The anti-hypertensive effect was achieved by acupuncture manipulations, and the anti-hypertensive effect of twirling reducing manipulation on spontaneously hypertensive rats was better than that of twirling uniform reinforcing-reducing and twirling reinforcing manipulations. (2) After regional homogeneity and amplitude of low-frequency fluctuations analyses, the hypothalamus, the brain region related to blood pressure, was activated in the twirling uniform reinforcing-reducing manipulation group; the corpus callosum and cerebellum were activated in the twirling reinforcing manipulation group; and the hypothalamus, olfactory bulb, corpus callosum, brainstem, globus pallidum, and striatum were activated in the twirling reducing manipulation group. (3) According to the functional connectivity analysis, different acupuncture manipulations increased the functional connections between seed points and the brainstem, olfactory bulb, and cerebellum, etc.

**Conclusion:**

These results suggest that acupuncture manipulations achieved the hypotensive effect and the twirling reducing manipulation had a better hypotensive effect on spontaneously hypertensive rats than twirling uniform reinforcing-reducing and twirling reinforcing manipulations; the central mechanism of the anti-hypertensive effect of twirling reinforcing and reducing manipulation may be related to the activation of brain regions associated with blood pressure regulation and the functional connections between them. Furthermore, brain regions involved in motor control, cognition, and hearing were also activated. We hypothesize that activation of these brain regions may help prevent or mitigate the onset and progression of hypertensive brain damage.

## 1. Introduction

Essential hypertension (EH) is a chronic, clinically frequent, and common disease affecting approximately 116 million adults in the United States and over 1 billion global adults. Although it is one of the leading causes of morbidity and mortality in the cardiovascular system, there is no definitive cure for this disease (Malpas, 2010; Gu et al., 2015; Nishijima et al., 2015; Yang et al., 2017; Carey et al., 2022). The number of adults with high blood pressure is estimated to reach 1.5 billion worldwide by 2025; this situation poses an increasingly serious challenge to global public health, thus treatment of hypertension has become a key issue that needs to be addressed (Kearney et al., 2005). Studies have found that a 10 mmHg reduction in systolic blood pressure can decrease the risk of cardiovascular disease by about 20%–30% (Malpas, 2010). Clinically, hypertension is mainly treated with drugs, including thiazides and angiotensin-converting enzyme inhibitors (Carey et al., 2022). These drugs have certain limitations, such as dependence, higher costs, and adverse effects (Calhoun et al., 2014; Oparil and Schmieder, 2015; Goshgarian and Gorelick, 2019; Flack and Adekola, 2020; Reeve et al., 2020). Numerous clinical and experimental studies have proven that acupuncture is an efficient complementary alternative therapy for reducing high blood pressure (Wang et al., 2018; Yang et al., 2022; Zhang et al., 2022). Compared with drug therapy, acupuncture to lower blood pressure does not cause adverse reactions associated with drug therapy, such as side effects of drugs, meanwhile reducing dependence on antihypertensive drugs and reducing the cost of medication, making it an effective option for controlling hypertension and its complications. In traditional Chinese medicine, the Taichong point (LR 3) is considered an infusion point and the original point of the Foot Turk’s Yin Liver meridian, which regulates the meridians, pacifies the liver, submerges the yang, and treats liver yang hyperactivity. Modern studies clearly established the unique advantages and efficacy of acupuncture at the “Taichong” point in decreasing blood pressure (Lu et al., 2015; Luo et al., 2019; Zhang et al., 2021a). One study found that acupuncture at LR 3 led to a rapid onset of action in treating hypertension by reducing systolic and diastolic blood pressure within 20 min (Han and Liang, 2021). Acupuncture twirling reinforcing and reducing manipulation (TRRM) is a key factor in the efficacy of acupuncture for decreasing blood pressure. The twirling uniform reinforcing-reducing (TUR) manipulation, twirling reinforcing (TRF) manipulation and twirling reducing (TRD) manipulation are the basic manipulations of TRRM. TUR is the twisting of the needle forward and backward with the same force after the needle is pierced into the skin at a certain depth; TRF means that after piercing the needle into the skin to a certain depth, the thumb applies a forward or backward twisting force to the needle and is heavier in the forward direction and lighter in the backward direction; TRD is a lighter force with the thumb forward and a heavier force with the thumb backward. TRRM is a treatment method based on the theory of the Nei Jing: treating excess with expelling, and treating deficiency with reinforcement, which means that if the evil Qi is strong and the positive Qi is not yet weakened, acupuncture can be used to treat by reducing, and if the positive Qi is weak and the body is poor, acupuncture can be used to treat by reinforcement. TRRM can achieve the multitarget regulation of blood pressure via the neuroendocrine system, cytokines, signal transduction pathways, and other pathways (Lu et al., 2017; Liang et al., 2021; Wu et al., 2021). However, the mechanisms underlying its anti-hypertensive effects remain unclear.

Functional magnetic resonance imaging (fMRI) is a common visualization technique used to reveal local brain functions (Zeng et al., 2012; Liu et al., 2014; Ko et al., 2016; Zhang et al., 2021b). Owing to its advantages of high temporal and spatial resolution, lack of radiation, fast imaging speed, and noninvasive nature, fMRI has become the most generally used neuroimaging technique in the study of the central mechanisms of acupuncture and the brain’s response to acupuncture stimulation (Kim et al., 2020; Huang et al., 2022). Resting-state fMRI (rs-fMRI), based on a blood oxygenation level-dependent (BOLD) technique, provides a wide variety of methods and tools for studying functional brain imaging metrics. Regional homogeneity (ReHo) is an imaging metric that describes the local functional activity of the brain and is used to evaluate the temporal synchronization and co-ordination of neuronal activity in the local brain areas (Zhang et al., 2019; Shan et al., 2020). Increased ReHo indicates the convergence of neuronal synchronization in local brain areas, whereas increased ReHo indicates dyssynchronization of nerve element activity. The amplitude of low-frequency fluctuation (ALFF) is an amplitude analysis of the low-frequency band of the BOLD signal, which is a reflection of the level of spontaneous activity of each voxel at rest from an energy point of view (Liang et al., 2014; Wang et al., 2016). An increase in ALFF value indicates increased excitability in that brain region, whereas a decrease in ALFF value suggests that decreased neurons’ excitability in that brain region (Yang et al., 2018). ALFF and ReHo deliver different information about different types of neuronal activity and are complementary methods ways to study changes in the whole brain. The combination of ALFF and ReHo may be a more comprehensive assessment of the pathophysiology of brain dysfunction than any of the approaches on their own (Li et al., 2018). Seed-point-based functional connectivity (FC) involves the analysis of the overall functional activity of the brain to determine whether FC exists between brain regions in a time series. The above three analytical methods are widely used in acupuncture to study the central mechanisms (Fang et al., 2015; Zhang et al., 2018, 2021a,b; Sun et al., 2021).

**Figure 1:**
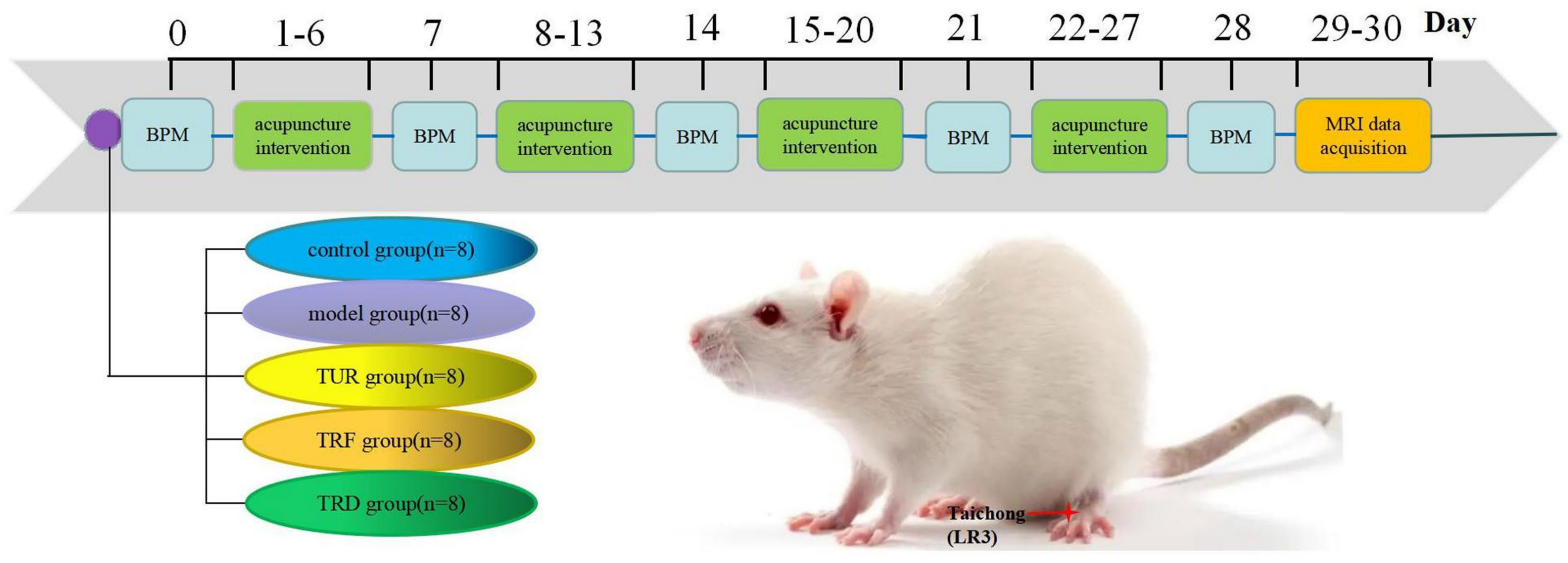
Experimental flowchart. TUR, twirling uniform reinforcing-reducing; TRF, twirling reinforcing; TRD, twirling reducing; BPM, blood pressure measurement.

Using positron emission computed tomography, we found that the hypotensive effect of acupuncture with TRRM could be exerted by increasing glucose metabolism in different target brain regions (Guo et al., 2018). However, the central regulation of blood pressure is a complex process involving several brain regions (Chen et al., 2013), and the central mechanism of TRRM in decreasing blood pressure remains unclear. Different brain imaging techniques use different principles and can reflect neurophysiological and pathological mechanisms from different perspectives. Therefore, the present study used rs-fMRI to assess the local brain function indices ReHo, ALFF, and FC in spontaneously hypertensive rats (SHRs) to elucidate other potential central mechanisms of blood pressure regulation by TRRM. This provides some objective scientific basis for the clinical application of the TRRM. Figure 1 shows a flowchart.

## 2. Materials and methods

### 2.1. Animal preparation

Thirty-two male SHRs at 12 weeks old and eight Wistar–Kyoto (WKY) rats at 12 weeks old were purchased from Beijing Vital River Laboratory Animal Technology Co., Ltd. in China (License No.SCXK (Jing)20210006). The experimental animals were housed according to specific standards for pathogen-free and supplied food and water *ad libitum* for 1 week. The rearing temperature was controlled throughout the study at 20 ± 1°C, and alternating light and dark lighting (12 h, 12 h) was used. All laboratory animal experiments were conducted in strict compliance with the World Health Organization International Guidelines for Biomedical Research Involving Animals and were approved by the Animal Care and Use Committee of Beijing University of Traditional Chinese Medicine (BUCM-4-2021040804-2140).

Thirty-two SHRs at 12 weeks old were randomly divided into four groups (*n* = 8/group): model group, twirling uniform reinforcing-reducing (TUR)manipulation group, twirling reinforcing (TRF) manipulation group, and twirling reducing (TRD) manipulation group. Eight WKY rats at 12 weeks old were used as control group.

### 2.2. Intervention methods

The LR 3 is located in the dorsal recess of the first and second metatarsal bones on the dorsal surface of the hind limbs. Before the operation, the rat was completely drilled into a comfortable soft mouse sleeve with its head fixed and both hind limbs exposed.

Needling intervention with the right hand as the stabbing hand was performed daily at 14:00 p.m. by a professional technician with the help of a metronome pronunciation frequency of 3 min at 60 times/min to facilitate the control of the twisting frequency and time. The duration of the intervention was 28 days (intermittently, 1 day per week, once per day).

In the TUR group, needles (0.18 mm × 13 mm, Beijing Zhongyan Taihe Medical Instrument Co., Ltd., Beijing, China) were inserted directly into LR 3 bilaterally for 1.5–2 mm. Twisting was then performed at a frequency of 60 times per minute within ±360° for 3 min, during which the thumb was twisted forward and backward with the same force. The needle retention time was 17 min.

In the TRF group, needles were inserted directly into LR 3 bilaterally for 1.5–2 mm. Twisting was then performed at a frequency of 60 times per minute within ±360° for 3 min, during which the thumb force forward was heavier and the thumb force backward was lighter. The needle retention time was 17 min.

In the TRD group, needles were inserted directly into LR 3 bilaterally for 1.5–2 mm. Twisting was performed at a frequency of 60 times per minute within ±360° for 3 min, during which the thumb force forward was lighter and the thumb force backward was heavier. The needle retention time was 17 min.

In the model and control groups, the rats were operated under the same constraints as the first three groups for 20 min of fixation without acupuncture treatment.

### 2.3. Blood pressure measurement

The rats were preheated at 37°C (constant temperature adjustment with thermostat) for approximately 2 min at room temperature of 22 ± 2°C, and the systolic blood pressure in the quiet and awake state was measured using a noninvasive blood pressure monitor (BP-2010E, Softron Biotechnology, Beijing, China). The blood pressure of each rat was measured three times, and the mean recorded blood pressure was used as the baseline value. Blood pressure was measured in all rats on day 0 (1 d before acupuncture) and on days 7, 14, 21, and 28. Blood pressure values were measured between 8:00 a.m. and 11:00 a.m. each day to keep errors to a minimum. The mesh and tail sleeve sizes should be appropriate during blood pressure measurements, and movements should be gentle to avoid fluctuations.

### 2.4. MRI data acquisition

A 7.0 T small animal live MRI scanner (PharmaScan 70/16 US, Bruker, Germany) with a dedicated small-animal cranial surface coil was used for scanning. Before scanning, each rat was injected intramuscularly with a dexmedetomidine hydrochloride injection (100 ug/mL) in the posterior lateral thigh at a dose of 0.02 mL per 100 g of rat body weight, and anesthesia was induced using a mixture of 5% isoflurane/95% oxygen. During the scan, the rat was placed in a prone position on a rat specific scanning platform, the rat’s head was held in place with dental hooks and ear sticks, and a 2% isoflurane/98% oxygen mixture was administered using a mask. First, T2-weighted sequences were scanned, followed by BOLD sequences. During the scanning process, a physiological detector monitored body temperature, respiration rate, and heart rate in real time (Model 1025, Small Animal Instruments Inc., Stony Brook, NY, United States). T2-weighted imaging was performed using T2-turboRARE sequence with the following parameters: repetition time (TR) = 5,500 ms; echo time (TE) = 33 ms; the number of averages = 2, slice thickness = 0.5 mm, percent phase field view = 100, acquisition matrix = 256 × 256; bandwidth = 152.587; and flip angle = 90°. BOLD-based rs-fMRI was also conducted using the T2Star_FID_EPI sequence with the following parameters: TR = 2,000 ms, TE = 11 ms, number of averages = 1, slice thickness = 0.5 mm, percent phase field view = 80, bandwidth = 2,500, acquisition matrix = 80 × 48, and flip angle = 90°.

### 2.5. Magnetic resonance data preprocessing and index calculation

Data preprocessing and metrics calculation were based on the Statistical Parametric Mapping (SPM) 12 package, REST package, FC toolkit, and the Data Processing & Analysis of Brain Imaging (DPABI) package of the MATLAB platform. The following procedures were used: (1) Format Conversion: Raw functional and T2 images were transformed from medical digital imaging and communication formats to the neuroimaging informatics initiative format. (2) Starting time point removal: When the functional image is acquired initially, the BOLD signal is often unstable; therefore, the first 10 time points must be removed. (3) Voxel augmentation: The SPM package is designed based on the actual size of the human brain, and the rat brain is much smaller than the human brain; therefore, the acquired rat MRI images need to be magnified 10 times to fit the package operation. (4) Slice timing: The data of all voxels were adjusted as if they were scanned simultaneously, thus making the acquisition time of all voxel BOLD signals theoretically consistent within a time point. (5) Realignment: A small amount of movement of each rat’s head was corrected between different time points in the scan. The exclusion criterion was a movement of more than one voxel size (magnified voxel size of 3 × 3 × 3 mm). (6) Reorientation: The average functional phase is homeopathically corrected, and the homeopathic correction matrix of the average functional phase is applied to the functional phase file; the T2 phase also needs to be corrected. (7) Normalization: The brains of all subjects were aligned to a uniform standard space to resolve the differences in brain shape between rats and the inconsistent spatial position of the head during scanning and to facilitate subsequent statistics. (8) Smooth: High-frequency noise is reduced from image deformation during spatial normalization and improves statistical validity; the smooth kernel size is two to three times the magnified voxel size. (9) Calculation of indicator: The REST and SPM12 toolkits must be loaded to calculate ALFF and ReHo. The calculation of ALFF was performed after the spatial smoothing, and ALFF was the selected range of 0.01–0.08 Hz. The calculation of ReHo was performed after the spatial normalization, and the noise in the frequency band below 0.01 Hz (low frequency) and above 0.08 Hz (high frequency) needed to be removed before ReHo calculation. The FC, SPM12, and DPABI toolkits must be loaded for the FC calculations. The calculation of FC was performed after the spatial smoothing.

### 2.6. Statistical analysis

#### 2.6.1. Systolic pressure data analysis

SPSS 20.0 statistical analysis software was used to process the blood pressure measurements. The data are presented as mean ± standard deviation, and the results were analyzed by two-way analysis of variance (ANOVA) for repeated measurements. Statistical significance was set at *p* < 0.05.

#### 2.6.2. fMRI data analysis

The magnetic resonance data were modeled using a general linear model. Data were analyzed using one-way ANOVA with a post-hoc two-sample *t*-test. Areas with significant ALFF and ReHo changes between the two groups were determined based on *p* < 0.005 (uncorrected) and clusters >5 voxels Areas with significant FC changes between the two groups were determined based on *p* < 0.005 (uncorrected) and clusters >2 voxels.

#### 2.6.3. Correlation analysis of systolic blood pressure and ReHo/ALFF

We performed a Pearson correlation analysis between systolic blood pressure and ReHo/ALFF values after TRRM. *p* < 0.05 was considered significant difference.

## 3. Results

### 3.1. Effects of TRRM on systolic blood pressure in SHRs

**Figure 2:**
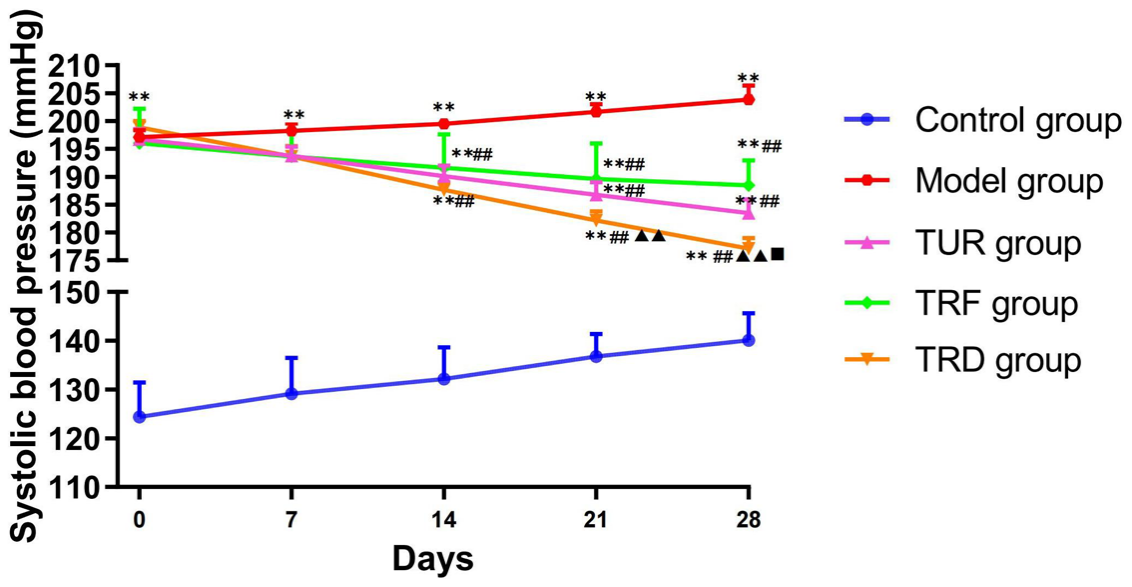
Evolution of systolic blood pressure measured in each group. All data are expressed as the mean ± SD (*n* = 8/group). ^**^p < 0.01 vs. control group; ^##^*p* < 0.01 vs. model group; ^▲▲^*p* < 0.01 vs. TRF group; ^■^*p* < 0.05 vs. TUR group. TUR, twirling uniform reinforcing-reducing manipulation; TRF, twirling reinforcing manipulation; TRD, twirling reducing manipulation.

The blood pressure of rats was significantly higher in model group on days 0, 7, 14, 21, and 28 of the experiment (*p* < 0.01) than in the control group, indicating that the hypertension data of the SHR model remained stable. Before the experiment, on day 0 of acupuncture, there was not statistically meaningful difference among the model, TUR, TRF, and TRD groups, suggesting that each group’s baseline blood pressure levels were consistent and comparable. On days 14th, 21st, and 28th days of acupuncture, the blood pressure of the SHRs in each acupuncture group was markedly below than that of the model group (*p* < 0.01). On the 21st and 28th days of acupuncture, compared with the TRF, the blood pressure of rats in the TUR group was no statistically meaningful between groups (*p* > 0.05), whereas the blood pressure of rats in the TRD group was markedly lower (*p* < 0.01). On the 28th day of acupuncture, the blood pressure of rats in the TRD group was significantly lower than that of rats in the TUR group (*p* < 0.05). This indicates that TRRM has a certain effect on decreasing blood pressure, and TRD has the most significant effect on decreasing blood pressure (Figure 2).

### 3.2. ReHo analysis

**Figure 3:**
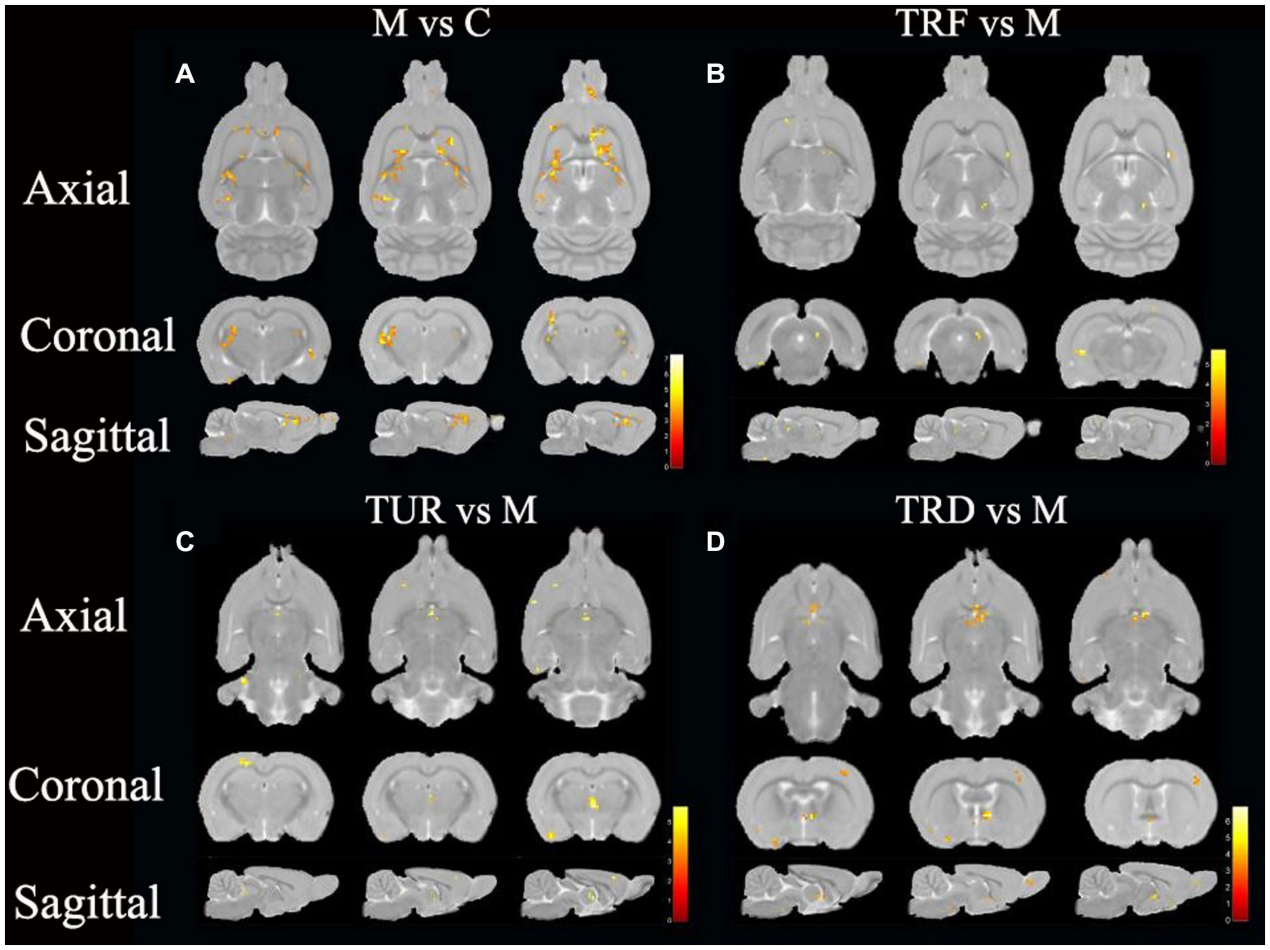
ReHo difference brain regions map. **(A)** Brain regions with decreased ReHo in the model group versus the control group (M vs. C); **(B)** Brain regions with increased ReHo in the TRF group versus the model group (TRF vs. M); **(C)** Brain regions with increased ReHo in the TUR group versus the model group (TUR vs. M); **(D)** Brain regions with increased ReHo in the TRD group versus the model group (TRD vs. M). The color bars were used to signify the *t*-value of the group analysis (the color is brighter; the *t*-value is higher).

The brain regions with decreased ReHo in the model group compared to that in the control group were the entorhinal cortex (left and right), olfactory bulb (left and right), hippocampal dentate gyrus (left), and brainstem (left and right), etc. (Figure 3A). The brain regions with increased ReHo in the TRF group compared to that in the model group were the brainstem (right), striatum (left), basal forebrain region (left), etc. (Figure 3B). The brain regions with increased ReHo in the TUR group compared to that in the model group were the hypothalamus (right), brainstem (right), primary visual cortex (right), etc. (Figure 3C). The brain regions with increased ReHo in the TRD group compared to that in the model group were the entorhinal cortex (left), primary motor cortex (left), etc. (Figure 3D). The locations of brain regions with significant differences are presented in Supplementary Table S1.

In this study, we focused on brain regions with a callback effect. We intersected brain regions with decreased ReHo values in the model group compared to that in the control group and brain regions with increased ReHo values in the treatment group compared to that in the model group to obtain the callback brain regions. These callback brain regions are critical brain regions for acupuncture intervention. Callback brain regions in the TUR group included the entorhinal cortex (left), hippocampal dentate gyrus (left and right), hypothalamus (left), etc. Callback brain regions in the TRF group included the brainstem (right), corpus callosum (right), cerebellum (right), basal forebrain region (left), etc. Callback brain regions in the TRD group included the entorhinal cortex (left), basal forebrain region (left), brainstem (left and right), descending corticofugal pathways and globus pallidum (left), hypothalamus (left), etc.

### 3.3. ALFF analysis

**Figure 4:**
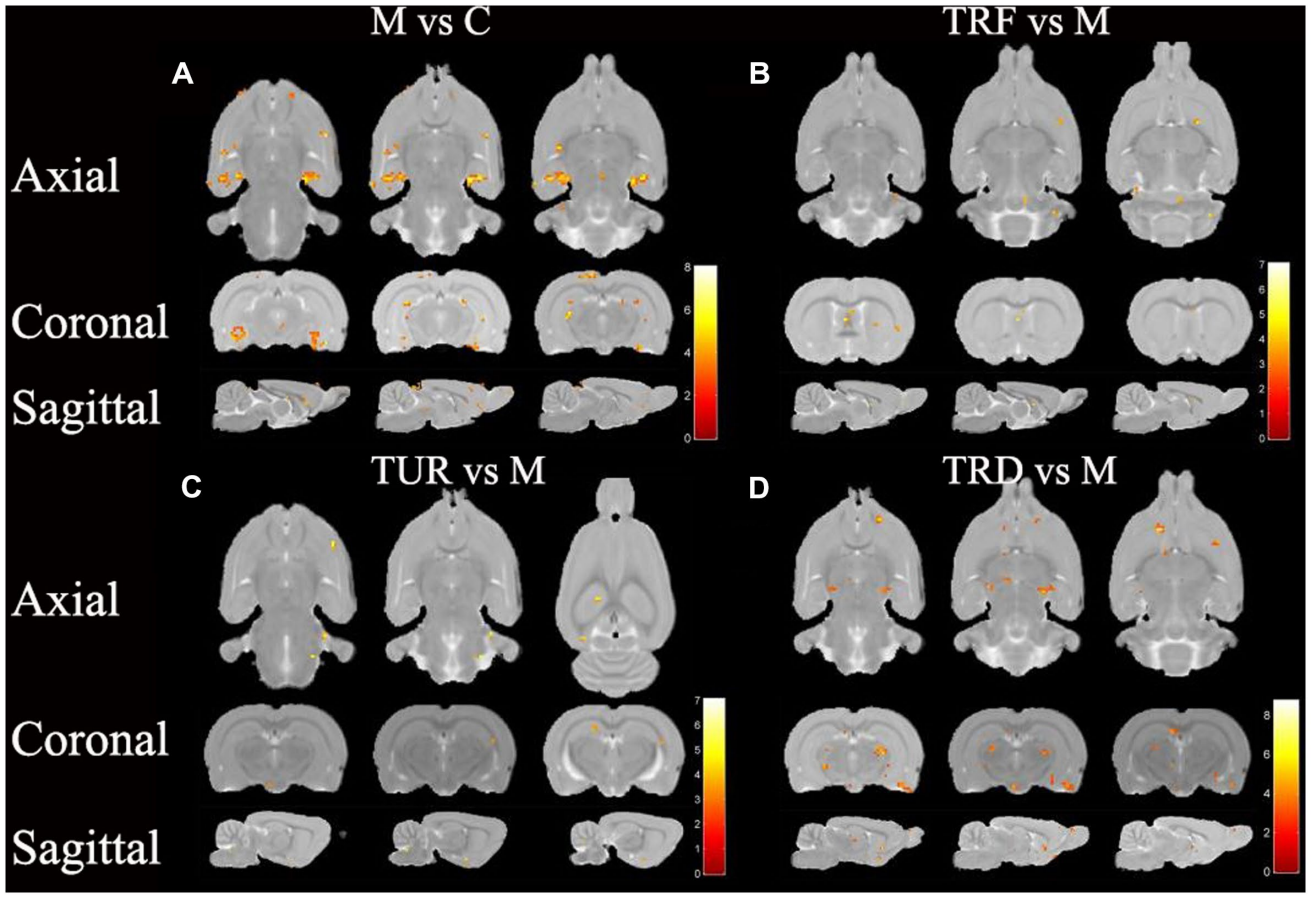
ALFF difference brain regions map. **(A)** Brain regions with decreased ALFF in the model group versus the control group (M vs. C); **(B)** Brain regions with increased ALFF in the TRF group versus the model group (TRF vs. M); **(C)** Brain regions with increased ALFF in the TUR group versus the model group (TUR vs. M); **(D)** Brain regions with increased ALFF in the TRD group versus the model group (TRD vs. M). The color bars were used to signify the *t*-value of the group analysis (the color is brighter; the *t*-value is higher).

The brain regions with decreased ALFF in the model group compared to that in the control group were the hypothalamus (left), entorhinal cortex (left and right), hippocampal dentate gyrus (left and right), etc. (Figure 4A). The brain regions with increased ALFF in the TRF group compared to that in the model group were the cerebellum (left and right), striatum (right), primary motor cortex (right), etc. (Figure 4B). The brain regions with increased ALFF in the TUR group compared to that in the model group were the hypothalamus (left), basal forebrain region (right), primary visual cortex (left), etc. (Figure 4C). The brain regions with increased ALFF in the TRD group compared to that in the model group were the thalamus (left and right), olfactory bulb (left and right), brainstem (left and right), etc. (Figure 4D). The locations of brain regions with significant differences are presented in Supplementary Table S2.

Similar to the ReHo analysis above, we focused on callback brain regions. Callback brain regions in the TUR group included the entorhinal cortex (right), corpus callosum (right), olfactory bulb (left), hypothalamus (left), etc. Callback brain regions in the TRF group included the entorhinal cortex (left), hippocampus (left), cerebellum (left and right), etc. Callback brain regions in the TRD group included the entorhinal cortex (left and right), hypothalamus (left), striatum (left and right), hippocampal dentate gyrus (left), etc.

### 3.4. Correlation analysis of systolic blood pressure and ReHo/ALFF

We intersected the callback brain regions based on ReHo analysis and the callback brain regions based on ALFF analysis to obtain common callback brain regions. These brain regions are usually the core brain regions for acupuncture intervention. The common callback brain regions in the TUR group included the hypothalamus (left) and hippocampal dentate gyrus (left). The common callback brain regions in the TRF group included the corpus callosum (right) and cerebellum (left and right). The common callback brain regions of the TRD group included the basal forebrain region (left), brainstem (right), descending corticofugal pathways and globus pallidum (left), striatum (right), hypothalamus (left), thalamus (left and right), preLimbic system (left), olfactory bulb (left), corpus callosum (left and right), secondary auditory cortex (left), primary somatosensory cortex forelimb (left and right), primary motor cortex (left), and entorhinal cortex (left).

**Figure 5:**
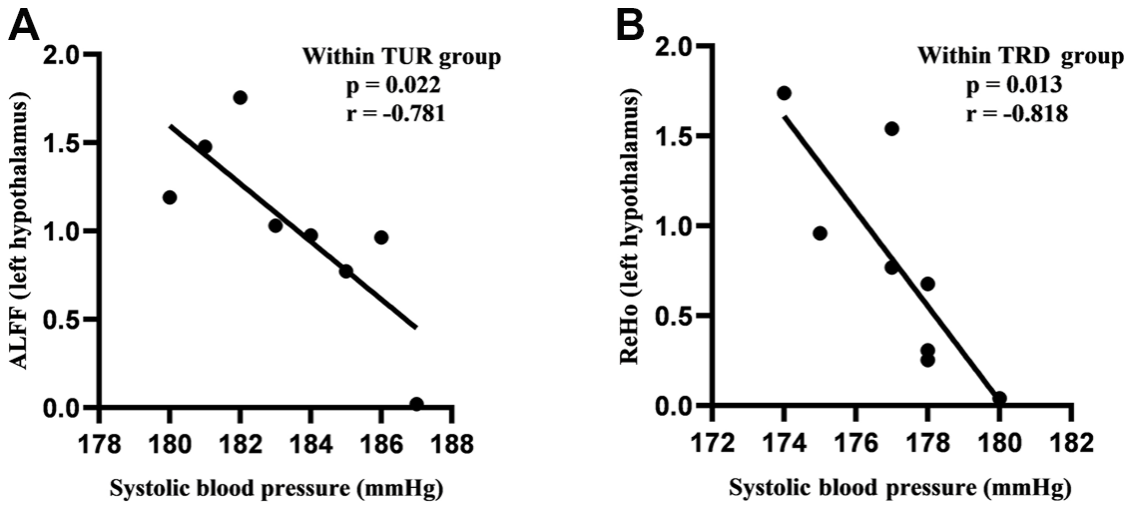
Correlation analysis of systolic blood pressure and ReHo/ALFF values. **(A)** Correlation analysis of systolic blood pressure and ALFF values in the TUR group. **(B)** Correlation analysis of systolic blood pressure and ReHo values in the TRD group. Pearson’s correlation was used with *p* < 0.05 for statistical significance and r for correlation.

The hypothalamus is closely related to blood pressure regulation. Based on the above ReHo/ALFF results, we found that the brain areas activated by TRRM included the left hypothalamus, we performed a Pearson correlation analysis between systolic blood pressure and ReHo/ALFF values of the left hypothalamus. The results revealed a negative correlation between systolic blood pressure and ALFF values in TUR group (*p* = 0.022, *r* = −0.781; Figure 5A), as well as a negative correlation between systolic blood pressure and ReHo values in TRD group (*p* = 0.013, *r* = −0.818; Figure 5B).

### 3.5. FC analysis

**Figure 6:**
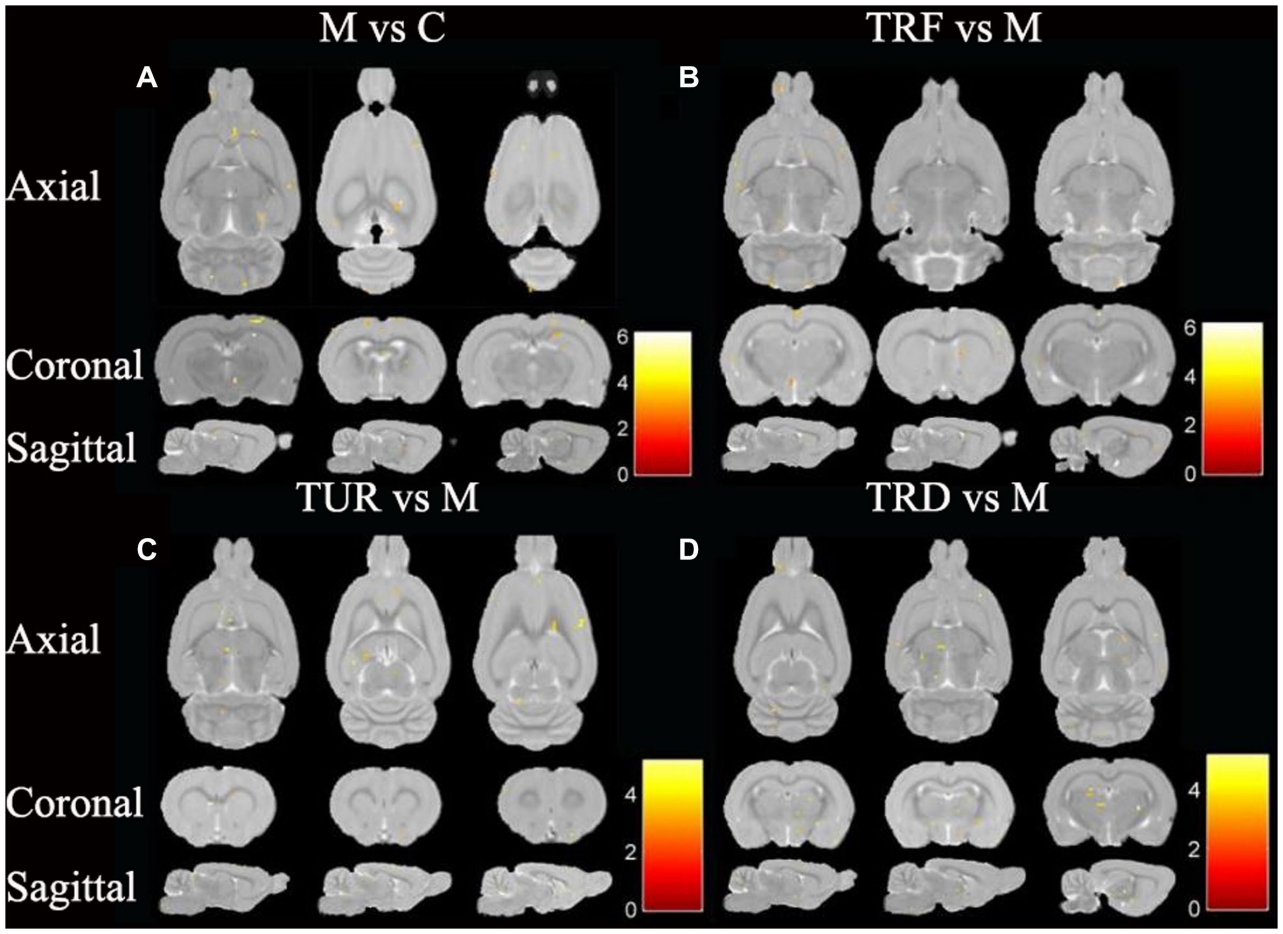
FC difference brain regions map. **(A)** Brain regions with decreased FC in the model group versus the control group (M vs. C); **(B)** Brain regions with increased FC in the TRF group versus the model group (TRF vs. M); **(C)** Brain regions with increased FC in the TUR group versus the model group (TUR vs. M); **(D)** Brain regions with increased FC in the TRD group versus the model group (TRD vs. M). The color bars were used to signify the *t*-value of the group analysis (the color is brighter; the *t*-value is higher).

Based on the results obtained by ReHo and ALFF, the left hypothalamus, a brain region closely related to blood pressure, was selected as the seed point and analyzed for FC in the whole brain to obtain differential brain regions. The brain regions with decreased FC in the model group compared to that in the control group were the brainstem (left and right), basal forebrain region (left and right), olfactory bulb (right), primary motor cortex (left and right), etc. (Figure 6A). Brain regions with increased FC in the TRF group compared to that in the model group were the thalamus (left), hippocampal dentate gyrus (left), etc. (Figure 6B). The brain regions with increased FC in the TUR group compared to that in the model group were the cerebellum (right), the primary somatosensory cortex (left and right), etc. (Figure 6C). The brain regions with increased FC in the TRD group compared to that in the model group were the cerebellum (left and right), thalamus (left and right), olfactory bulb (left and right), etc. (Figure 6D). The locations of brain regions with significant differences are presented in Supplementary Table S3.

Similar to the ReHo and ALFF analysis, we focused on the callback brain regions. Callback brain regions in the TUR group included the basal forebrain region (right), periaqueductal Gray (left), striatum (left), etc. Callback brain regions in the TRF group included the brainstem (left and right), Primary cingular cortex (left and right), olfactory bulb (left), etc. Callback brain regions in the TRD group included the primary somatosensory cortex (left and right), secondary motor cortex (left and right), entorhinal cortex (left), dentate gyrus (left and right), etc.

## 4. Discussion

Compared with the model groups, each treatment group showed a gradual anti-hypertensive effect from day 14, and the extent of the anti-hypertensive effect varied among the treatment groups. On days 14–28 of the acupuncture intervention, the difference between the anti-hypertensive effect of each treatment and the model group was stable. The TRD group had a significantly better anti-hypertensive effect than the TRF and TUR groups. There was no statistically significant difference between the TRF and TUR groups; however, the trend of systolic blood pressure decrease in the TUR group was more evident than in the former. Acupuncture manipulations decreased the blood pressure of the SHRs, and the hypotensive effect of TRD on the SHRs was better than that of TUR or TRF. This result is consistent with a previous study (Wu et al., 2021), in which acupuncture manipulations had different hypotensive effects.

In the rs-fMRI study, we combined ALFF and ReHo analysis to identify changes in the brain regions of SHRs, which do not depend on the effects of model-generated errors and can directly suggest the presence of spontaneous neuronal activity, which is reliable, practical, and sensitive for monitoring local brain function (Li et al., 2018). After ReHo and ALFF analysis, the callback brain regions associated with blood pressure regulation that were activated in the TUR group was the hypothalamus; the TRF group included the corpus callosum and cerebellum; the TRD group included the hypothalamus, olfactory bulb, corpus callosum, brainstem, globus pallidum, and striatum. The results showed extensive regional alterations in the brain regions of SHRs. However, each acupuncture intervention group reversed some of the alterations and activated different target brain regions related to blood pressure regulation, which may explain the different anti-hypertensive effects of TRRM. The central regulatory regions of the cardiovascular system are widely distributed and include the brainstem, medulla oblongata, periaqueductal gray matter of the midbrain, hypothalamus, and amygdala (Chen et al., 2013). Some of these brain regions are the key target areas involved in central blood pressure regulation, forming a complete network. Using positron emission tomography for brain imaging, a previous study found that acupuncture with TRRM significantly reduced blood pressure in SHRs with clear central effects and achieved central regulation of blood pressure by increasing glucose metabolism levels in several target brain regions, including the hypothalamus, medulla oblongata, hippocampus, and cerebellum, similar to the activated brain regions in our findings (Guo et al., 2018).

The hypothalamus was activated in both the TUR and TRD groups. A previous study showed that the hypothalamus is the neuroendocrine center, and endocrine disorders lead to elevated cortisol levels, which is significantly associated with increased blood pressure (Gold et al., 2005). The hypothalamic supraoptic nucleus and paraventricular nucleus secrete pressor hormone and oxytocin, which act on the pituitary system and interact with neurons of cardiovascular activity (Qin et al., 2018). It has been found that acupuncture with the supraoptic and paraventricular nuclei as seed points strengthens the functional connections between the hypothalamus and the frontal lobes, cerebellum, and insula, suggesting that these brain regions constitute a neural network structure with specific functions and elaborating the mechanism of its anti-hypertensive effects (Zheng et al., 2016). Acupuncture for EH in the hypothalamic-associated resting brain network has revealed that acupuncture can regulate the cardiovascular system via the intricate brain networks of the cortex, hypothalamus, and brainstem. After performing FC analysis using the left hypothalamus as the seed point, our results showed that different acupuncture manipulations enhanced the FC of the left hypothalamus with the brainstem, olfactory bulb, and cerebellum and that these brain regions overlapped with the blood pressure–callback brain regions activated by ReHo and ALFF analysis, which may further indicate that these brain regions play a key role in blood pressure regulation.

The brainstem is the brain region activated by TRD, and FC analysis has revealed enhanced FC between the brainstem and hypothalamus. It is located at the core of the central nervous system and has a crucial function in regulating elevated EH (Cheng et al., 2020). The current study demonstrated that the nucleus tractus solitarius and reactive oxygen species are essential factors in the neural mechanisms of hypertension by increasing central sympathetic outflow and inhibiting the pressure reflex regulation of blood pressure via their effects on the lateral ventral and nucleus tractus solitarius of the medulla oblongata (Chan and Chan, 2014). And Cheng found abnormal insulin signaling pathways in the brainstem of SHRs during hypertension, which is a potentially important indicator of a lack of brainstem metabolic disorders (Cheng et al., 2020). The pathology of hypertension involves immune, metabolic, and neuroendocrine processes and provides new insights into the dysregulation of the central nervous system associated with metabolite levels (Van Arendonk et al., 2023). The cerebellum is a specific brain region that TRD activates to maintain balance and coordinate random movements; however, its synergistic relationship with other brain regions is not fully understood. The cerebellar adrenomedullin (AM) is involved in regulating blood pressure. Dysregulation of cerebellar earthworm AM, its receptor components, and AM signaling pathway occurs during hypertension (Figueira and Israel, 2017). Connections between the cerebellum and the seed points may reveal the cerebellum’s role in blood pressure regulation (Zheng et al., 2016).

Both the olfactory bulb and striatum, which are extensions of the telencephalon, were activated in the TRD group. It projects many nerve fibers to the hypothalamus. It is closely associated with the amygdala, pyriform nucleus, and hypothalamus’s ventral medial and posterior nuclei, which are involved in the regulation of cardiovascular function (Edwards et al., 1993). Significantly enhanced norepinephrine transmission in the asymmetric olfactory bulb of hypertensive rats may contribute to hypertension development, maintenance, and progression (Cassinotti et al., 2018). Guil supported the correlation between olfactory bulb regulation in chronically elevated blood pressure and the powerful effects of endothelin (Guil et al., 2019). Abnormalities in the brain’s dopamine system contribute to the progression of hypertension (Watanabe et al., 1997). Sawamura found high levels of dopamine in the striatum and that extracellular dopamine levels in the striatum correlated with variations in blood pressure, suggesting that dopamine in the striatum is engaged in the progression of 2 K-1C hypertension and that the striatum may be the region engaged in the development of hypertension (Sawamura and Nakada, 1996). We found that the TRD group co-activated the corpus callosum with the TUR group. In the traditional pathological sense, memory impairment and epilepsy often occur with corpus callosum lesions, and psychiatric abnormalities and limb dysfunction can occur. The corpus callosum has complex connections with the insula and limbic and paralimbic regions of the cerebral hemispheres (Shah et al., 2021). The microstructural integrity of the corpus callosum is associated with overall cognition, and appropriate blood pressure treatment may delay these changes and cause concomitant cognitive dysfunction (Gons et al., 2012). We found that the corpus callosum was activated, which may be an effect of the acupuncture technique to prevent further development of hypertension and slow the development of cerebral small vessel disease.

In the correlation analysis, we found a high negative correlation between blood pressure and brain function after acupuncture intervention, indicating that TRRM can activate the functions of brain regions related to blood pressure regulation and play an important role in lowering blood pressure.

This study has some limitations. First, we explored the central mechanism of TRRM in decreasing blood pressure from the perspective of rs-fMRI; however, we did not conduct deeper molecular mechanism research. In the future, we will investigate the relevant mechanisms from a multi-omics perspective. Second, we used only one index, systolic blood pressure, to assess changes in blood pressure in rats. Multiple indices should be used in future studies to obtain a more objective evaluation. Third, under the premise of meeting the experimental requirements (FJalal et al., 2015; Chen et al., 2017,  2021; Li et al., 2018; Jiang et al., 2022), we referred to the relevant literature and only performed the fMRI scans of each group of rats after the experimental acupuncture intervention, and did not perform the fMRI scans of each group of rats before the acupuncture intervention, which might make the experimental data incomplete. In future experiments, we will perform brain scans of each group of rats at baseline to further improve the rigor of the experimental design.

## 5. Conclusion

Our results showed that TRD, TRF, and TUR decreased blood pressure, with TRD having a greater effect than TRF or TUR. The three acupuncture treatments activated different target brain regions related to blood pressure regulation and enhanced the functional connection between the hypothalamus and brain regions relevant to blood pressure. This finding clarifies how the three acupuncture manipulations produce different anti-hypertensive effects. In addition, brain regions involved in vision, motor control, cognition, and hearing were activated. Activation of these regions may help prevent or reduce the development and progression of hypertensive brain damage and its complications.

## Data availability statement

The original contributions presented in the study are included in the article/Supplementary material, further inquiries can be directed to the corresponding authors.

## Ethics statement

The animal study was reviewed and approved by the Institutional Animal Care and Use Committee of the Beijing University of Chinese Medicine (BUCM-4-2021040804-2140).

## Author contributions

Y-YL participated in the experimental procedures and wrote, and revised the manuscript. J-PL participated in blood pressure measurements and graphing of the article. S-FS and K-ZY translated the manuscript. YG, JS, QX, and X-LW analyzed the data. Q-GL and MX designed and directed the experiment participated in reviewing and revising the article and provided funding. All authors contributed to the article and approved the submitted version.

## Funding

This study was funded by the National Natural Science Foundation of China (No. 82074553) and the Fundamental Research Fund of Beijing University of Traditional Chinese Medicine (No. 2021-JYB-XJSJJ-084).

## Conflict of interest

The authors declare that the research was conducted in the absence of any commercial or financial relationships that could be construed as a potential conflict of interest.

## Publisher’s note

All claims expressed in this article are solely those of the authors and do not necessarily represent those of their affiliated organizations, or those of the publisher, the editors and the reviewers. Any product that may be evaluated in this article, or claim that may be made by its manufacturer, is not guaranteed or endorsed by the publisher.
