# Supplementary material for: Acupuncture with twirling reinforcing and reducing manipulation shows a control of hypertension and regulation of blood pressure-related target brain regions in spontaneously hypertensive rat: a preliminary resting-state functional MRI study
Source: Front Neurosci. 2023 May 26;17:1161578. doi: 10.3389/fnins.2023.1161578 (PMC10250630; doi:10.3389/fnins.2023.1161578)
Supplement: Supplementary file 1 [file Data_Sheet_1.PDF]

Supplementary Material

Acupuncture withTwirlingReinforcing andReducing Manipulation Shows a Control of Hypertension andRegulation ofBlood Pressure-RelatedTargetBrainRegionsin Spontaneously HypertensiveRats: A PreliminaryResting-State Functional MRI Study

Supplementary Table1 : ReHo analysis of differential brain regions

Table 1A : Specific localization of the differential brain regions with decreased ReHo in the model group (M)vs. control group(C)

M vs C↓	brain regions	Voxel size	t-value	MNI peak point coordinates		
				X（mm）	Y（mm）	Z（mm）
	Entorhinal cortex_L	7	-3.4214	-58	-26.05	-29.8
	Amygdalopiriform cortex_L	14	-5.593	-58	-38.05	-26.8
	Entorhinal cortex_R	6	-4.4532	44	-23.05	-20.8
	Subiculum_L	16	-4.5901	-43	-50.05	-17.8
	Medial entorhinal cortex_R<br>Subiculum_R<br>Perirhinal area 36_R	51	-5.547	50	-59.05	-14.8
	Lateral entorhinal cortex internal part_R	15	-4.673	65	-62.05	-14.8
	Brainstem_L<br>Hypothalamic region_L	8	-4.3973	-10	-56.05	-5.8
	Lateral entorhinal cortex_L	5	-5.062	-73	-50.05	-8.8
	Posterior agralunar insular cortex_L	6	-3.7263	-70	-20.05	-8.8
	Brainstem_R	7	-4.5114	14	-98.05	-2.8
	striatum_R	19	-4.8327	59	-20.05	3.2
	Anterior commissure anterior part_L<br>Olfactory bulb_L	6	-3.9867	-19	33.95	0.2
	Descending corticofugal pathways and globus pallidum_L<br>Primary somatosensory cortex forelimb_L<br>Thalamus_L<br>Fimbria of the hippocampus2_L	204	-6.707	-40	0.95	39.2
	Periventricular grey_L	7	-4.6099	-7	-89.05	9.2
	Agranular insular cortex_L	10	-4.4975	-37	27.95	18.2
	Cornu ammonis3_R	19	-4.3753	47	-44.05	12.2
	Cornu ammonis3_L	5	-4.3838	-46	-47.05	12.2
	striatum_L	9	-4.1987	-25	24.95	18.2

Corpus callosum and associated subcortical white matter_R	5	-3.4968	50	-20.05	24.2
Cornu ammonis1_L	7	-4.1966	-61	-56.05	24.2
Descending corticofugal pathways and globus pallidum_R	12	-5.5042	44	-20.05	15.2
PreLimbic system_L	6	-5.275	-4	21.95	18.2
Dentate gyrus_L	14	-5.8484	-49	-56.05	24.2
Descending corticofugal pathways and globus pallidum_R	14	-4.4747	38	-20.05	27.2
Fimbria of the hippocampus2_R	13	-5.2747	26	-14.05	24.2
Olfactory bulb_R	26	-4.6909	8	72.95	27.2
Primary motor cortex_L	21	-6.1228	-31	18.95	51.2
Primary somatosensory cortex jaw_L	6	-4.8814	-40	30.95	27.2
PreLimbic system_R<br>Primary motor cortex_R	7	-3.9249	23	48.95	39.2
secondary auditory cortex dorsal part_L	5	-4.2653	-58	-59.05	39.2
Corpus callosum and associated subcortical white matter_R	40	-5.9425	38	-11.05	42.2
Primary cingular cortex_L	25	-5.1515	-1	27.95	45.2
Orbitofrontal region_R	14	-4.7607	38	51.95	36.2
Orbitofrontal region_L	17	-7.0982	-34	45.95	36.2
Lateral secondary visual cortex_L<br>Primary visual cortex binocular area_L	5	-4.5931	-52	-65.05	42.2
Granule cell level of the cerebellum_L<br>Granule cell level of the cerebellum_R<br>Molecular layer of the cerebellum_R	6	-4.0423	5	-107.05	9.2
Primary somatosensory cortex forelimb_R	7	-5.062	38	9.95	60.2
Corpus callosum and associated subcortical white matter_L	9	-5.3392	-40	-23.05	42.2

Abbreviations : L, left; R, right; ↓, decreased.

Table 1B : Specific localization of the differential brain regions with increased ReHo in the TRF group (B)vs. model group(M).

B vs M ↑	brain regions	Voxel size	t-value	MNI peak point coordinates		
				X（mm）	Y（mm）	Z（mm）
	Brainstem_R	5	4.3389	20	-116.05	-29.8
	Perirhinal area 36_L	5	3.6219	-46	-74.05	-8.8
	Striatum_L	5	5.5751	-19	9.95	-8.8
	Basal forebrain region_L	8	4.6517	-31	-2.05	-5.8
	Molecular layer of the cerebellum_R	6	4.4389	11	-134.05	3.2
	Cornu ammonis3_L	7	5.322	-52	-47.05	12.2
	Thalamus_R	5	4.0521	23	-14.05	18.2
	Deeper layers of the superior colliculus_R	8	4.295	20	-68.05	27.2
	Corpus callosum and associated subcortical white matter_R	7	5.7198	50	-11.05	27.2
	Primary visual cortex monocular area_L	10	4.2269	-22	-89.05	45.2
	Medio lateral secondary visual cortex_R	5	5.1424	32	-50.05	54.2

Abbreviations : L, left; R, right; ↑, increased.

Table 1C : Specific localization of the differential brain regions with increased ReHo in the TUR group (P)vs. model group(M)

P vs M ↑	brain regions	Voxel size	t-value	MNI peak point coordinates		
				X（mm）	Y（mm）	Z（mm）
	Entorhinal cortex_L	11	3.7729	-49	-20.05	-26.8
	Hypothalamic region_R<br>Hypothalamic region_L	6	4.0135	2	-47.05	-11.8
	Lateral Entorhinal cortex external part_L	10	4.8133	-49	-62.05	-11.8
	Middle cerebellar peduncle_R	9	3.8237	35	-80.05	-8.8
	Brainstem_R	6	3.3976	26	-86.05	-5.8
	Molecular layer of the cerebellum_L	9	4.6349	-46	-98.05	0.2
	Basal forebrain region_L	9	5.2601	-1	-14.05	3.2
	Dentate gyrus_R<br>Dentate gyrus_L	14	5.5154	2	-20.05	6.2
	Agranular insular cortex_L<br>PreLimbic system_L	5	4.3235	-40	21.95	9.2
	Perirhinal area 36_L<br>Medial entorhinal cortex_L	10	3.9271	-61	-83.05	9.2
	Secondary somatosensory cortex_L	7	4.6462	-64	0.95	9.2
	Granule cell level of the cerebellum R	5	4.6336	32	-119.05	18.2
	Periaqueductal gray_L	8	5.5248	-4	-77.05	18.2
	Deeper layers of the superior colliculus_R	7	4.7797	23	-71.05	30.2
	Cornu ammonis1_R	6	4.6636	41	-38.05	42.2
	Primary cingular cortex_L	8	4.6046	-1	30.95	48.2
	Medial parietal associative cortex_L<br>Lateral parietal associative cortex_L	15	4.6174	-22	-29.05	57.2
	Primary visual cortex monocular area_R	7	5.0843	23	-86.05	57.2

Abbreviations : L, left; R, right; ↑, increased.

Table 1D : Specific localization of the differential brain regions with increased ReHo in the TRD group (X)vs. model group(M).

X vs M ↑	brain regions	Voxel size	t-value	MNI peak point coordinates		
				X（mm）	Y（mm）	Z（mm）
	Entorhinal cortex_L	9	4.7035	-31	-11.05	-29.8
	Descending corticofugal pathways and globus pallidum_L	27	6.5124	-7	-74.05	-23.8
	Brainstem_R	8	3.642	20	-38.05	-14.8
	Substantia nigra_L	14	4.6735	-10	-56.05	-5.8
	Basal forebrain region_R	5	3.9206	5	24.95	-8.8
	Perirhinal area 36_L	9	4.3722	-46	-65.05	-8.8
	Hypothalamic region_L Thalamus_L	6	3.9873	-7	-14.05	-2.8
	Basal forebrain region_L Bed nucleus of the stria terminalis_R	29	6.7974	14	-5.05	3.2
	Medial entorhinal cortex_L Lateral entorhinal cortex_L	5	4.3015	-61	-83.05	9.2
	PreLimbic system_L Olfactory bulb_L	7	3.9633	-37	42.95	12.2
	Brainstem_L Periaqueductal gray_L	5	3.2151	-7	-71.05	9.2
	Secondary somatosensory cortex_L	5	4.9459	-64	-20.05	15.2
	Thalamus_R	10	4.3682	20	-14.05	15.2
	Primary somatosensory cortex barrel field_R Primary somatosensory cortex dysgranular_R	22	4.926	50	6.95	42.2
	Primary somatosensory cortex barrel field_L	6	3.7942	-55	-38.05	39.2
	Corpus callosum and associated subcortical white matter_L	12	5.308	-55	-29.05	27.2
	Olfactory bulb_R	17	4.5741	8	72.95	27.2
	Primary motor cortex_L	23	4.408	-28	12.95	51.2
	Medio lateral secondary visual cortex_R Corpus callosum and associated subcortical white matter_R Primary visual cortex_R	12	6.8487	32	-50.05	54.2

Abbreviations : L, left; R, right; ↑, increased.

Supplementary Table2 : ALFF analysis of differential brain regions

Table 2A : Specific localization of the differential brain regions with decreased ALFF in the model group (M)vs. control group(C).

M vs C↓	brain regions	Voxel size	t-value	MNI peak point coordinates		
				X（mm）	Y（mm）	Z（mm）
	Hypothalamic region_L	8	-6.7166	-19	-29.05	-23.8
	Basal forebrain region_L	7	-4.4588	-10	0.950012	-23.8
	Amygdalopiriform cortex_L	5	-3.9608	-46	-59.05	-20.8
	Dentate gyrus_R<br>Perirhinal area 35_R<br>Subiculum_R<br>Parasubiculum_R<br>Entorhinal cortex_R	146	-6.5478	41	-62.05	0.2
	Amygdalopiriform cortex_R	7	-5.4473	56	-56.05	-14.8
	Entorhinal cortex_L	24	-7.0737	-55	-26.05	-8.8
	Subiculum_L<br>Perirhinal area 35_L<br>Parasubiculum_L	144	-6.3658	-58	-62.05	3.2
	Perirhinal cortex_R<br>Lateral entorhinal cortex_R	7	-4.5949	71	-35.05	-5.8
	Olfactory bulb_L	9	-5.345	-10	45.95	-8.8
	Descending corticofugal pathways and globus pallidum_L	42	-5.1074	-46	-26.05	3.2
	Posterior agralunar insular cortex_R<br>Dysgranular insular cortex_R	9	-6.4712	62	-11.05	-2.8
	Lateral entorhinal cortex_L	9	-4.8835	-76	-68.05	3.2
	Anterior commissure anterior part_R	5	-3.8858	20	33.95	-2.8
	Brainstem_R	7	-3.9075	5	-59.05	3.2
	Granule cell level of the cerebellum_L	10	-4.3171	-22	-101.05	12.2
	Olfactory bulb_R	29	-6.0582	11	48.95	12.2
	Cornu ammonis3_R	5	-5.1455	50	-53.05	15.2
	Perirhinal area 36_R	7	-3.4961	62	-83.05	18.2
	Thalamus_R	10	-4.7487	38	-68.05	18.2
	Thalamus_L	16	-5.7211	-40	-47.05	21.2
	PreLimbic system_L<br>secondary cingular cortex_L<br>Corpus callosum and associated subcortical white matter_L	134	-7.1355	-7	18.95	30.2
	PreLimbic system_R	12	-5.0894	32	45.95	18.2

Molecular layer of the cerebellum_R	6	-4.3929	29	-92.05	21.2
striatum_R	9	-3.8778	41	-8.05	21.2
Perirhinal cortex_L Presubiculum_L	7	-3.8016	-67	-86.05	21.2
Dentate gyrus_L	6	-4.1886	-37	-65.05	24.2
Corpus callosum and associated subcortical white matter_R	5	-3.5849	53	-17.05	21.2
Thalamus_R	14	-7.1896	38	-38.05	30.2
Descending corticofugal pathways and globus pallidum_R	7	-5.3446	32	-5.05	24.2
Ventral hippocampal commissure_L	5	-4.4422	-1	-11.05	30.2
Glomerular layer of the olfactory bulb_L	5	-3.2803	-25	90.95	27.2
Cornu ammonis3_L	9	-4.9472	-40	-53.05	33.2
Secondary motor cortex_R	17	-7.8548	14	51.95	36.2
Molecular layer of the cerebellum_L Retosplenial dysgranular cortex_L	31	-4.9395	-25	-92.05	39.2
Orbitofrontal region_L	15	-4.5735	-34	57.95	33.2
Primary somatosensory cortex forelimb_R	31	-8.004	32	-5.05	45.2
secondary motor cortex_L	7	-3.6448	-13	54.95	36.2
Molecular layer of the cerebellum_L	12	-4.2805	-43	-122.05	45.2
Primary cingular cortex_R	9	-3.8318	5	27.95	48.2
Primary motor cortex_R	6	-4.1811	23	45.95	45.2
Frontal association cortex_R	5	-3.5109	29	57.95	45.2
Retosplenial dysgranular cortex_R External cortex of the Inferior colliculus_R	31	-4.969	5	-86.05	51.2
Primary motor cortex_L	8	-6.0367	-28	48.95	48.2
Primary somatosensory cortex forelimb_L	6	-4.5648	-49	24.95	51.2
Primary somatosensory cortex hindlimb_R	7	-4.1132	32	-5.05	66.2

Abbreviations : L, left; R, right; ↓, decreased.

Table 2B : Specific localization of the differential brain regions with increased ALFF in the TRF group (B)vs. model group(M).

B vs M ↑	brain regions	Voxel size	t-value	MNI peak point coordinates		
				X（mm）	Y（mm）	Z（mm）
	Amygdalopiriform cortex_L	5	4.4209	-55	-38.05	-23.8
	Cornu ammonis1_R<br>Entorhinal cortex_R	7	3.6434	41	-41.05	-14.8
	Middle cerebellar peduncle_R	11	3.7388	44	-95.05	0.2
	Dysgranular insular cortex_R	5	3.7494	56	0.95	9.2
	Granule cell level of the cerebellum_R	5	4.4855	47	-116.05	12.2
	Molecular layer of the cerebellum_R	7	3.8719	8	-98.05	12.2
	Molecular layer of the cerebellum_L	6	3.5689	-43	-86.05	18.2
	striatum_R	6	4.8309	32	-2.05	12.2
	Fimbria of the hippocampus2_L<br>Fornix_L	5	5.1127	-4	0.95	18.2
	Olfactory bulb_L	8	7.0287	-7	63.95	21.2
	Septal region_R	5	4.4455	-2	-2.05	27.2
	Orbitofrontal region_R<br>Primary motor cortex_R	8	5.63	35	42.95	39.2
	Olfactory bulb_R	5	3.9767	8	84.95	45.2

Abbreviations : L, left; R, right; ↑, increased.

Table 2C : Specific localization of the differential brain regions with increased ALFF in the TUR group (P)vs. model group(M).

P vs M ↑	brain regions	Voxel size	t-value	MNI peak point coordinates		
				X（mm）	Y（mm）	Z（mm）
	Entorhinal cortex_R	10	6.963	26	-14.05	-29.8
	Hypothalamic region_L<br>Mammillothalamic Tract_L	5	3.4818	-7	-47.05	-17.8
	Basal forebrain region_R	6	5.1726	32	-14.05	-17.8
	Agranular insular cortex_R	8	4.9601	53	9.95001	-2.8
	Inferior cerebellar peduncle n<br>Spinal trigeminal tract_R	5	4.8185	32	-122.05	0.2
	Molecular layer of the cerebellum_R	7	5.0166	44	-95.05	0.2
	Granule cell level of the cerebellum_R	5	4.5633	38	-122.05	9.2
	Corpus callosum and associated subcortical white matter R	7	3.692	56	-38.05	27.2
	Olfactory bulb_L	5	3.5401	-10	102.95	27.2
	Primary visual cortex binocular area_L	7	4.784	-37	-80.05	42.2
	Cornu ammonis1_L<br>Dentate gyrus_L	7	4.7605	-22	-32.05	45.2

Abbreviations : L, left; R, right; ↑, increased.

Table 2D : Specific localization ofthe differential brain regions with increased ALFF in the TRD group (X)vs. model group(M).

X vs M ↑	brain regions	Voxel size	t-value	MNI peak point coordinates		
				X（mm）	Y（mm）	Z（mm）
	Amygdalopiriform cortex_R	32	4.4824	59	-44.05	-20.8
	Entorhinal cortex_R	11	4.2026	35	-8.05	-26.8
	Mammillothalamic tract_L<br>Hypothalamic region_L	6	4.37	-4	-44.05	-20.8
	Cornu ammonis1_R<br>Cornu ammonis3_R	8	3.3468	38	-47.05	-8.8
	Entorhinal cortex_L	18	4.5476	-55	-23.05	-14.8
	Olfactory bulb_L	6	3.799	-16	42.95	-11.8
	Olfactory bulb_R	15	5.6445	26	33.95	0.2
	Brainstem_R	25	5.3248	32	-53.05	3.2
	Brainstem_L	9	4.6842	-34	-47.05	3.2
	Periaqueductal gray_L	5	3.908	-10	-41.05	3.2
	Bed nucleus of the stria terminalis_L	5	4.2859	-13	-2.05	9.2
	Dorsal fornix_L<br>striatum_L	26	8.7129	-19	21.95	9.2
	Dentate gyrus_L	5	3.5642	-40	-53.05	12.2
	Striatum_R	6	4.7021	50	9.95	6.2
	Thalamus_R	42	5.9543	32	-50.05	24.2
	PreLimbic system_R	5	4.2397	11	27.95	18.2
	Primary somatosensory cortex upperlips_L	6	4.2704	-55	-23.05	21.2
	Fimbria of the hippocampus3_R	5	4.0718	23	-8.05	21.2
	Primary somatosensory cortex Jaw_R	8	4.3142	41	30.95	27.2
	Thalamus_L	7	3.8517	-31	-44.05	21.2
	Glomerular layer of the olfactory bulb_L	5	3.4307	-25	87.95	21.2
	Septal region_R	7	3.7077	2	3.95	27.2
	PreLimbic system_R	8	4.2481	14	33.95	33.2
	Orbitofrontal region_R	11	4.3161	44	39.95	33.2
	Primary somatosensory cortex dysgranular_R	14	3.5911	50	15.95	48.2
	Corpus callosum and associated subcortical white matter_L<br>Cornu ammonis1_L	20	4.7411	-4	-32.05	39.2
	Primary somatosensory cortex dysgranular_L<br>cortex forelimb_L	19	5.545	-40	36.95	51.2

	Primary motor cortex_R	11	3.8026	32	18.95	48.2
	Primary somatosensory cortex barrel field_L	11	4.4091	-40	-5.05	48.2
	Primary motor cortex_L secondary motor cortex_L	6	4.6502	-28	48.95	48.2

Abbreviations : L, left; R, right; ↑, increased.

Supplementary Table3 : FC analysis of differential brain regions

Table 3A : Specific localization of the differential brain regions with decreased FC in the model group (M)vs. control group(C).

M vs C ↓	brain regions	Voxel size	t-value	MNI peak point coordinates		
				X（mm）	Y（mm）	Z（mm）
	Brainstem_R	2	-3.3033	8	-101.05	-32.8
	Brainstem_L	3	-3.4262	-19	-134.05	-29.8
	Basal forebrain region_R	2	-3.838	14	18.95	-26.8
	Basal forebrain region_L	2	-3.4602	-25	-11.05	-20.8
	Substantia nigra_R	2	-3.183	8	-50.05	-5.8
	Agranular insular cortex_L<br>Entorhinal cortex_L	2	-3.7668	-55	6.95	-5.8
	Perirhinal area 35_L	2	-3.4248	-43	-65.05	-2.8
	Periaqueductal gray_L	2	-3.2862	-1	-68.05	3.2
	Descending corticofugal pathways and globus pallidum_R	3	-4.3213	38	-20.05	3.2
	PreLimbic system_L	3	-3.5541	-31	42.95	9.2
	Agranular insular cortex_R	3	-4.0126	47	33.95	12.2
	Secondary somatosensory cortex_R	4	-4.3026	56	6.95	18.2
	PreLimbic system_R	5	-6.1393	5	39.95	18.2
	Molecular layer of the cerebellum_R	2	-3.7146	17	-143.05	21.2
	Thalamus_R	2	-3.4216	35	-65.05	21.2
	Primary auditory cortex_R	2	-4.0741	68	-29.05	21.2
	Olfactory bulb_L	2	-3.5185	-22	78.95	21.2
	Primary somatosensory cortex upperlips_L	2	-3.9611	-70	-23.05	27.2
	Secondary auditory cortex dorsal part_R	2	-3.7632	68	-26.05	27.2
	Primary somatosensory cortex upperlips_R	2	-3.7382	74	-20.05	27.2
	Ventral hippocampal commissure_R<br>Dentate gyrus_R	3	-3.3732	2	-8.05	27.2
	Striatum_L	2	-3.1194	-37	3.95	30.2
	Granule cell level of the cerebellum_R	2	-3.5992	38	-113.05	30.2
	Granule cell level of the cerebellum_L	2	-3.9637	-19	-122.05	36.2
	Cornu ammonis3_R	2	-4.8078	32	-50.05	33.2
	Orbitofrontal region_R	3	-4.8007	41	48.95	36.2

Primary cingular cortex_R	3	-3.108	17	36.95	36.2
Subiculum_R	2	-3.2038	32	-56.05	42.2
Deeper layers of the superior colliculus_R<br>Retosplenial dysgranular cortex_R	2	-3.3425	20	-77.05	45.2
Primary somatosensory cortex forelimb_R	3	-4.1063	38	3.95	45.2
Molecular layer of the cerebellum_L	4	-3.6968	-13	-146.05	51.2
Primary visual cortex binocular area_L	2	-3.4539	-46	-68.05	48.2
Cornu ammonis1_R<br>Corpus callosum and associated subcortical white matter_R	5	-5.4257	29	-44.05	48.2
Primary somatosensory cortex Dysgranular_R	2	-3.918	47	21.95	48.2
Primary somatosensory cortex barrel field_L	4	-3.1421	-58	-8.05	54.2
Secondary motor cortex_R<br>Primary cingular cortex_R	3	-3.5813	14	9.95	51.2
Primary motor cortex_L	2	-4.5415	-22	21.95	54.2
Secondary motor cortex_L	7	-4.0623	-16	-11.05	63.2
Primary somatosensory cortex hindlimb_R	2	-3.2746	32	0.94999<br>7	60.2
Primary visual cortex_R	2	-4.1031	53	-47.05	63.2
Medio lateral secondary visual cortex_R<br>Medio medial secondary visual cortex_R	6	-4.7248	35	-44.05	63.2
Primary cingular cortex_L	2	-3.3605	-4	-11.05	63.2
Primary motor cortex_R	2	-3.3605	23	-8.05	66.2

Abbreviations : L, left; R, right; ↓, decreased.

Table 3B : Specific localization of the differential brain regions with increased FC in the TRF group (B)vs. model group(M).

B vs M	brain regions	Voxel	t-value	MNI peak point coordinates		
↑		size		X（mm）	Y（mm）	Z（mm）
	Inferior olive n pyramidaldecusation n SP5_L	2	3.4954	11	-107.05	-29.8
	Hypothalamic region_L	2	3.3078	-4	-26.05	-29.8
	Lateral entorhinal cortex external part_R Perirhinal area 36_R	2	3.1448	53	-65.05	-8.8
	Brainstem_R	2	3.7295	29	-77.05	-2.8
	Middle cerebellar peduncle_L	2	4.2806	-46	-95.05	3.2
	Olfactory bulb_R	2	4.4274	8	45.95	0.199997
	Periaqueductal gray_L	2	3.8601	-1	-68.05	3.2
	Dentate gyrus_L	2	4.1468	-58	-56.05	6.2
	Molecular layer of the cerebellum_L	2	4.8894	-4	-92.05	12.2
	Molecular layer of the cerebellum_R	4	3.2511	17	-146.05	12.2
	Granule cell level of the cerebellum_L	2	3.3615	-13	-107.05	12.2
	Brainstem_L	2	3.4673	-16	-71.05	15.2
	Primary auditory cortex_L	2	3.6965	-64	-29.05	15.2
	secondary somatosensory cortex_L	2	3.8702	-67	-5.05	15.2
	secondary somatosensory cortex_R	4	3.6212	59	12.95	15.2
	Agranular insular cortex_R	2	3.5647	41	33.95	18.2
	Olfactory bulb_L	3	3.7558	-13	84.95	15.2
	Cornu ammonis3_L	2	3.4819	-49	-44.05	18.2
	Primary somatosensory cortex dysgranular_R	2	3.802	41	-2.05	39.2
	Corpus callosum and associated subcortical white matter_R	2	3.316	23	3.95	39.2
	Primary somatosensory cortex upperlips_R	2	4.657	56	3.95	39.2
	Retosplenial dysgranular cortex_R	2	3.7054	41	-89.05	42.2
	Lateral secondary visual cortex_L	2	3.4357	-61	-53.05	48.2
	Retrosplenial granular cortex	3	3.8137	-4	-59.05	48.2

part A_L<br>Retrosplenial granular cortex<br>part B_L					
striatum_R	3	3.7831	20	6.95	18.2
Primary cingular cortex_L<br>Primary cingular cortex_R	2	3.6668	-1	-8.05	54.2
Retosplenial dysgranular cortex_L	3	6.3458	-1	-23.05	57.2
Retrosplenial granular cortex part B_R	5	4.9967	2	-32.05	60.2

Abbreviations : L, left; R, right; ↑, increased.

Table 3C : Specific localization of the differential brain regions with increased FC in the TUR group (P)vs. model group(M).

P vs M ↑	brain regions	Voxel size	t-value	MNI peak point coordinates X（mm）	Y（mm）	Z（mm）
	Hypothalamic region_R	2	3.982	11	-23.05	-32.8
	Brainstem_L	2	3.8208	-25	-119.05	-26.8
	Brainstem_R<br>Brachium pontis_R	3	5.4412	20	-80.05	-26.8
	Entorhinal cortex_L<br>Amygdalopiriform cortex_L	9	4.2815	-49	-41.05	-23.8
	Inferior cerebellar peduncle n spinal trigeminal tract_L	2	3.4944	-37	-131.05	-17.8
	Hypothalamic region_L	4	4.1308	5	-20.05	-11.8
	Entorhinal cortex_R	2	3.1972	53	0.949997	-11.8
	Basal forebrain region_R	2	3.8838	32	0.949997	-11.8
	Molecular layer of the cerebellum_L<br>Middle cerebellar peduncle__L	4	3.7826	-40	-98.05	-2.8
	Lateral entorhinal cortex_L	2	3.8336	-73	-80.05	-5.8
	Periventricular grey_R	3	3.4042	11	-137.05	-2.800003
	Inferior cerebellar peduncle n<br>Spinal trigeminal tract_R	2	3.3296	38	-128.05	-2.800003
	Molecular layer of the cerebellum_R	3	3.5354	47	-104.05	0.1999969
	Descending corticofugal pathways and globus pallidum_R	2	3.1161	29	-11.05	0.199997
	Olfactory bulb_R	2	3.1538	32	33.95	0.199997
	Periaqueductal gray_L	4	4.5172	-1	-68.05	6.2
	Dentate gyrus_R	2	3.265	44	-59.05	6.2
	Agranular dysgranular insular cortex_R<br>Agranular insular cortex_R	4	3.7776	44	42.95	12.2
	Granule cell level of the cerebellum_L	2	3.2418	-13	-110.05	15.2
	Fimbria of the hippocampus2_L<br>Septal region_L	2	3.7974	-4	3.95	15.2
	Olfactory bulb_L	2	3.6147	-16	51.95	12.2
	Thalamus_L	2	3.7393	-7	-32.05	15.2
	Striatum_L	2	4.1894	-13	12.95	18.2

Secondary auditory cortex ventral part_L	3	4.2371	-64	-59.05	18.2
Deeper layers of the superior colliculus_R	2	3.3452	14	-53.05	21.2
Corpus callosum and associated subcortical white matter_L<br>Cornu ammonis3_L<br>Cornu ammonis2_L	4	3.6722	-55	-41.05	30.2
Granule cell level of the cerebellum_R	2	3.4325	41	-113.05	30.2
Striatum_R	2	3.1766	20	18.95	30.2
PreLimbic system_L	2	2.9963	-7	30.95	30.2
Dentate gyrus_L	3	3.1868	-28	-38.05	33.2
PreLimbic system_R	2	3.1443	11	39.95	33.2
Primary somatosensory cortex Jaw_L<br>Primary somatosensory cortex dysgranular zone 0_L	3	4.0984	-52	30.95	36.2
Frontal association cortex_R<br>secondary motor cortex_R	2	3.4784	5	51.95	39.2
External cortex of the inferior colliculus_L	2	3.8227	-19	-92.05	39.2
Corpus callosum and associated subcortical white matter_R	4	3.5358	23	-8.05	39.2
Primary somatosensory cortex barrel field_R<br>Primary somatosensory cortex upperlips_R	6	4.4918	56	-2.05	39.2
Superficial gray layer of the superior colliculus_L	2	3.9415	-13	-62.05	42.2
Lateral secondary visual cortex_R<br>parietal cortex postero rostral_R	2	3.5311	59	-50.05	42.2
Lateral parietal associative cortex_L	2	4.4078	-46	-35.05	45.2
Primary cingular cortex_R	2	3.9783	2	0.949997	54.2
Lateral parietal associative cortex_R<br>Medial parietal associative cortex_R	2	3.5041	26	-29.05	60.2
Primary motor cortex_L	4	3.6121	-16	-8.05	60.2
secondary motor cortex_L	2	3.7131	-13	21.95	63.2

Abbreviations : L, left; R, right; ↑, increased.

Table 3D : Specific localization of the differential brain regions with increased FC in the TRD group (X)vs. model group(M).

X vs M ↑	brain regions	Voxel size	t-value	MNI peak point coordinates		
				X（mm）	Y（mm）	Z（mm）
	Brachium pontis_R	2	3.3873	8	-86.05	-35.8
	Inferior olive n pyramidaldecusation n SP5_L	2	3.5432	11	-107.05	29.8
	Entorhinal cortex_R	4	3.8241	56	-20.05	-26.8
	Dentate gyrus_L	2	3.7316	-28	-44.05	-17.8
	Fornix_L	2	3.5831	-10	-35.05	-14.8
	Hypothalamic region_R Fornix_R	4	3.6374	14	-14.05	-17.8
	Entorhinal cortex_L	2	4.563	-61	-8.05	-17.8
	Brainstem_L	2	4.2638	-10	-98.05	-11.8
	Descending corticofugal pathways and globus pallidum_L Thalamus_L	2	3.5423	-19	-17.05	-5.8
	Basal forebrain region_R	2	3.5921	14	-2.05	-8.8
	Cornu ammonis3_R	2	3.9385	41	-50.05	0.199997
	Molecular layer of the cerebellum_R	2	4.0277	26	-119.05	3.199997
	Granule cell level of the cerebellum_L	3	4.9978	-40	-98.05	12.2
	Corpus callosum and associated subcortical white matter_L Lateral entorhinal cortex_L	2	4.1514	-58	-74.05	6.2
	Brainstem_R	2	4.5772	32	-62.05	6.2
	Thalamus_R	3	5.7493	35	-32.05	12.2
	Periventricular grey_R	2	3.4044	14	-83.05	12.2
	Deeper layers of the superior colliculus_R	2	4.5615	20	-53.05	12.2
	Agranular insular cortex_R	3	4.2724	41	33.95	15.2
	Periaqueductal gray_L	3	3.5763	-7	-32.05	15.2
	Primary auditory cortex_L	2	3.5228	-64	-29.05	18.2
	striatum_L	2	3.781	-34	-5.05	18.2
	Agranular insular cortex_L Dysgranular insular cortex_L	2	3.1	-52	33.95	18.2
	Molecular layer of the cerebellum_L	2	3.4787	-7	-140.05	21.2
	Primary somatosensory cortex Barrel field_R Primary somatosensory cortex	2	4.4708	65	-14.05	24.2

Upperlips_R					
Primary auditory cortex_R	2	3.3513	74	-62.05	27.2
Dentate gyrus_R	2	3.3981	26	-20.05	24.2
Olfactory bulb_R	2	3.8541	23	57.95	27.2
Primary somatosensory cortex dysgranular Zone 0_L<br>Primary somatosensory cortex upperlips_L	2	3.1026	-52	21.95	30.2
Fasciola cinereum_L	2	3.477	-10	-41.05	30.2
Primary somatosensory cortex Jaw_L	2	3.6255	-34	30.95	30.2
Retosplenial dysgranular cortex_R	3	3.397	41	-89.05	36.2
Frontal association cortex_R	2	3.9643	29	60.95	36.2
Olfactory bulb_L	2	3.4817	-16	72.95	36.2
Primary motor cortex_R	2	3.8086	20	45.95	45.2
Frontal association cortex_L	2	3.078	-16	63.95	45.2
Primary cingular cortex_R	3	4.2485	14	36.95	51.2
secondary motor cortex_R	3	5.2253	14	9.95	54.2
Medio lateral secondary visual cortex_R<br>Primary visual cortex monocular area_R	2	3.9819	26	-56.05	66.2
Primary motor cortex_L<br>secondary motor cortex_L	2	4.7425	-19	-29.05	69.2

Abbreviations : L, left; R, right; ↑, increased.
